# Bilateral Ampiginous Choroiditis following Presumed SARS-CoV-2 Infection

**DOI:** 10.1155/2021/1646364

**Published:** 2021-08-05

**Authors:** Elysse S. Tom, K. Matthew McKay, Steven S. Saraf

**Affiliations:** Department of Ophthalmology, University of Washington, Seattle, WA, USA

## Abstract

**Purpose:**

To report a case of bilateral ampiginous choroiditis following presumed SARS-CoV-2 infection. *Case Description*. A 25-year-old woman presented with metamorphopsia and a paracentral scotoma in her left eye. She endorsed night sweats, headache, and new-onset anosmia beginning 1 week before her visual symptoms. She also had multiple confirmed ill COVID-19 contacts at her workplace before the onset of her symptoms. Funduscopic examination and multimodal imaging revealed placoid lesions in the macula and midperiphery of both eyes consistent with ampiginous choroiditis. COVID-19 antibody testing returned positive for IgG, and an extensive systemic evaluation was otherwise unremarkable. She was treated with oral prednisone and azathioprine with stabilization of the retinal lesions and no progression of her symptoms.

**Conclusions:**

Ampiginous choroiditis is an inflammatory chorioretinopathy with an unknown pathogenic mechanism that often necessitates early immunomodulatory therapy. This report suggests that SARS-CoV-2 infection may trigger chorioretinal inflammation in susceptible hosts.

## 1. Introduction

Severe Acute Respiratory Syndrome Coronavirus-2 (SARS-CoV-2), the novel coronavirus that causes COVID-19, is associated with a multitude of adverse systemic effects beyond acute respiratory disease. Molecular mimicry has been proposed as a contributing factor to some systemic complications, including antiphospholipid antibodies, Kawasaki disease, and Guillain-Barre syndrome. Systemic corticosteroids have shown benefit in some patients [1, 2].

Ampiginous choroiditis is an inflammatory chorioretinopathy with features of both acute posterior multifocal placoid pigment epitheliopathy (APMPPE), an often self-limited condition with good prognosis, and serpiginous choroiditis, a progressive condition with high risk of visual disability. Both can affect otherwise healthy individuals and have been hypothesized to occur due to molecular mimicry between infectious and ocular antigens. APMPPE is often preceded by a flu-like illness, and cases have been associated with vaccinations, as well as infections with group A streptococcus, adenovirus type 5, tuberculosis, Lyme disease, and mumps [3–5].

## 2. Case Report

**Figure 1:**
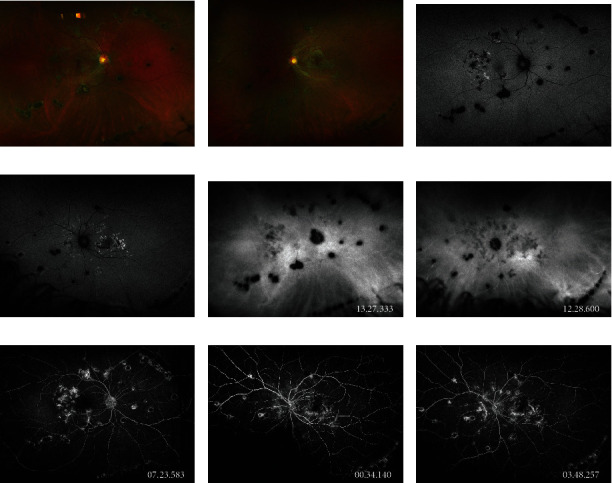
Multimodal fundus imaging. Optos fundus photos of the (a) right and (b) left eyes demonstrating placoid lesions in the macula and retinal periphery as well as peripheral punched-out scars in a Schlaegel line configuration. (c, d) Fundus autofluorescence exhibits hypoautofluorescent placoid lesions with irregular hyperautofluorescent borders in the posterior pole and midperiphery of both eyes. (e, f) Indocyanine green angiography displays hypofluorescent placoid choroidal lesions more widespread in the nasal periphery of the left eye than visible on fundus autofluorescence, signifying active inflammatory disease. Fluorescein angiography of the (g) right eye and (h) left eye early and (i) late demonstrates progressive staining and leakage from the placoid lesions.

**Figure 2:**
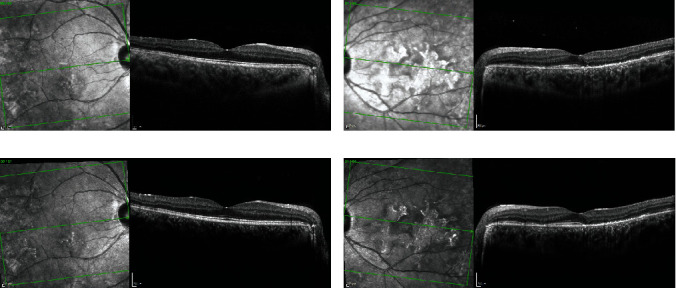
Optical coherence tomography. Optical coherence tomography of the (a) right and (b) left eyes on initial visit. The macular lesions are associated with outer retinal irregularities in both eyes. After initiating treatment, the placoid lesions in the (c) right and (d) left eyes did not progress or improve 8 weeks after the initial visit.

A 25-year-old healthy woman presented complaining of distortion and a blind spot near the central vision of her left eye of one month duration. One week preceding visual symptom onset, she endorsed night sweats, headache, and new-onset anosmia. The night sweats and headaches lasted 3 days. The anosmia lasted 2 weeks. She had multiple confirmed ill COVID-19 contacts at her workplace before the onset of her symptoms. Best corrected visual acuity was 20/20 and 20/25 in the right and left eyes, respectively. Intraocular pressures, previously documented as normal, were 20 mmHg on the right and 24 mmHg on the left after one month of topical difluprednate therapy. The anterior chambers were both quiet with no cell or flare. Examination of the vitreous revealed rare cell and no haze in both eyes. Dilated fundus examination revealed placoid lesions in the macula and midperiphery of both eyes. Peripheral punched-out scars with Schlaegel lines were also noted. Fundus imaging (Figure 1) demonstrated hypoautofluorescent lesions in the macula and midperiphery of both eyes with irregular hyperautofluorescent borders. Hypofluorescent lesions were observed on indocyanine green angiography in a distribution more widespread in the left eye than appreciated on fundus examination. Fluorescein angiography (FA) demonstrated early progressive staining and leakage from the placoid lesions without significant early blockage. Optical coherence tomography demonstrated bilateral outer retinal disruption (Figure 2). Laboratory evaluation was negative for QuantiFERON-TB gold, HIV, and syphilis antibodies. COVID-19 antibody testing returned positive for IgG. COVID-19 PCR testing was not performed. The patient's systemic symptoms had occurred 4 months following the initial pandemic lockdowns in the United States when the patient's county of origin had reported a total of 450-460 cases per 100,000 inhabitants [6]. Neuroimaging was not pursued due to the resolution of headaches and absence of neurologic symptoms at the time of evaluation. She was treated with a taper of oral prednisone starting at 60 mg. The retinal lesions did not progress, and steroid-sparing therapy with azathioprine (1.5 mg/kg) was initiated at 3 weeks. Visual acuity remained 20/20 in both eyes at 10-month follow-up after completing a 3-month oral prednisone taper and continuing azathioprine.

## 3. Discussion

Ampiginous choroiditis is an inflammatory chorioretinopathy with high risk of visual disability that shares disease features with APMPPE and serpiginous choroiditis [7]. The disease pathogenesis is unknown, but similar processes have been observed to occur following flu-like illnesses as well as specific viral entities [3]. We present a case of ampiginous choroiditis shortly following presumed SARS-CoV-2 infection demonstrated by positive antibody testing.

To our knowledge, this is the second report of posterior uveitis potentially associated with COVID-19 . Serpiginous choroiditis was recently reported 2 weeks after onset of systemic symptoms in a case of PCR-proven COVID-19 [8]. In addition to the new serpiginous choroiditis lesions, there was documentation of preexisting peripapillary findings that could have represented an earlier manifestation of the disease. In the case of our patient, without positive PCR testing, recent SARS-CoV-2 infection was presumed based on systemic symptom onset one week prior to the development of subjective vision changes and five weeks prior to positive antibody testing. Minimally symptomatic patients may demonstrate COVID-19 antibodies for only 6 weeks after infection [9], supporting a recent primary infection in our patient, rather than a more remote, asymptomatic infection.

Differentiating APMPPE from ampiginous choroiditis is critical due to implications for prognosis and treatment. While APMPPE is often a self-limited condition with good prognosis, ampiginous choroiditis is characterized by recurrent inflammation with high risk for permanent vision loss [7]. The presence of smaller, more numerous, nonhealing lesions extending into the peripheral retina supported a diagnosis of ampiginous choroiditis in this case. Furthermore, the active lesions in the left eye did not demonstrate the classic early blocking of fluorescence on FA observed in APMPPE. The diagnosis of serpiginous-like choroiditis was also considered given the nonhealing nature of the lesions, but this possibility was less favored given negative tuberculosis testing and lack of evidence of prominent edge reactivation. Consideration of these features and the presence of fovea-threatening lesions led to the initiation of immunomodulatory therapy. Local steroids were avoided due to demonstrated steroid response and cataract risk in a young patient. Intraocular pressures normalized after topical difluprednate was discontinued, and pachymetry revealed thick corneas so further glaucoma testing was not pursued.

As reported in the serpiginous choroiditis case by Providência et al., we observed evidence of prior ocular inflammatory disease before the COVID-19-associated flare [8]. We observed inactive peripheral punched-out scars in each eye possibly associated with a history of multifocal choroiditis or ocular histoplasmosis syndrome. Multifocal choroiditis with panuveitis has been reported to overlap with other white dot syndromes [10]. Our patient had features of both active ampiginous choroiditis and chronic findings consistent with a multifocal choroiditis. It is possible that COVID-19 could serve as an immunologic trigger for the development or reactivation of ocular inflammatory disease in susceptible hosts, in this case in a patient with a propensity for overlapping white dot syndromes.

## 4. Conclusions

Ampiginous choroiditis is an inflammatory chorioretinopathy with unknown pathogenic mechanism that often necessitates early immunomodulatory therapy. This report suggests that SARS-CoV-2 infection may trigger chorioretinal inflammation in susceptible hosts.

## Data Availability

The data used to support the findings of this study are available from the corresponding author upon request.

## References

[1] Angileri F., Legare S., Marino Gammazza A., Conway de Macario E., Jl Macario A., Cappello F. (2020). Molecular mimicry may explain multi-organ damage in COVID-19. *Autoimmunity Reviews*.

[2] Ehrenfeld M., Tincani A., Andreoli L. (2020). Covid-19 and autoimmunity. *Autoimmunity Reviews*.

[3] Borruat F. X., Piguet B., Herbort C. P. (1998). Acute posterior multifocal placoid pigment epitheliopathy following mumps. *Ocular Immunology and Inflammation*.

[4] Gonome T., Suzuki Y., Metoki T., Takahashi S., Nakazawa M. (2016). Acute posterior multifocal placoid pigment epitheliopathy and granulomatous uveitis following influenza vaccination. *American Journal of Ophthalmology Case Reports*.

[5] Dutta Majumder P., Biswas J., Gupta A. (2019). Enigma of serpiginous choroiditis. *Indian Journal of Ophthalmology*.

[6] (2021). COVID-19 Data Dashboard. https://www.doh.wa.gov/Emergencies/COVID19/DataDashboard#reports.

[7] Jones B. E., Jampol L. M., Yannuzzi L. A. (2000). Relentless placoid chorioretinitis: a new entity or an unusual variant of serpiginous chorioretinitis?. *Archives of Ophthalmology*.

[8] Providência J., Fonseca C., Henriques F., Proença R. (2020). Serpiginous choroiditis presenting after SARS-CoV-2 infection: a new immunological trigger?. *European Journal of Ophthalmology*.

[9] Ibarrondo F. J., Fulcher J. A., Goodman-Meza D. (2020). Rapid Decay of Anti–SARS-CoV-2 Antibodies in Persons with Mild Covid-19. *New England Journal of Medicine*.

[10] Kuznetcova T., Jeannin B., Herbort C. P. (2012). A case of overlapping choriocapillaritis syndromes: multimodal imaging appraisal. *Journal of ophthalmic & vision research*.

